# 流式细胞术检测新诊断多发性骨髓瘤患者GPRC5D表达情况及其预后价值

**DOI:** 10.3760/cma.j.cn121090-20250220-00080

**Published:** 2025-04

**Authors:** 琮倩 金, 芬 颜, 嫒 马, 开林 徐, 洁云 夏

**Affiliations:** 徐州医科大学附属医院血液科，徐州 221002 Department of Hematology, the Affiliated Hospital of Xuzhou Medical University, Xuzhou 221002, China

**Keywords:** 多发性骨髓瘤, GPRC5D, 预后, Multiple myeloma, GPRC5D, Prognosis

## Abstract

**目的:**

观察GPRC5D在新诊断多发性骨髓瘤（NDMM）患者骨髓瘤细胞上的表达情况并探讨其预后价值。

**方法:**

回顾性分析2023年4月至2024年4月在徐州医科大学附属医院就诊的65例NDMM患者的临床资料。所有患者在诱导治疗前均应用流式细胞术检测骨髓瘤细胞表面GPRC5D的表达情况，根据GPRC5D阳性率中位数将患者分为GPRC5D高表达组和低表达组，比较两组患者的临床特征、免疫状态、诱导治疗后疗效及预后。

**结果:**

65例NDMM患者浆细胞GPRC5D阳性率的中位数为32.68％，以此为界值将患者分为GPRC5D高表达组（33例，GPRC5D阳性率≥32.68％）和GPRC5D低表达组（32例，GPRC5D阳性率<32.68％）。与GPRC5D低表达组相比，GPRC5D高表达组1q21扩增比例较高（78.8％对43.8％，*P*＝0.004），出现≥2种未受累免疫球蛋白免疫麻痹比例（87.9％对62.5％，*P*＝0.018）和重度免疫麻痹比例（59.4％对33.3％，*P*＝0.046）较高，CD16^+^CD56^+^细胞比例较低［（16.60±8.70）％对（27.78±15.78）％，*P*＝0.005］。GPRC5D高表达和低表达组诱导治疗的总体反应率差异无统计学意义（78.8％对93.8％，*P*＝0.165），但GPRC5D高表达组疗效达到非常好的部分缓解及以上的比例更低（42.4％对78.2％，*P*＝0.003），且微小残留病阴性率也较低（30.0％对68.8％，*P*＝0.002）。与GPRC5D低表达组相比，GPRC5D高表达组患者具有更短的中位无进展生存期（11.2个月对未达到，*P*＝0.002），两组患者的中位总生存期均未达到，差异无统计学意义（*P*＝0.069）。

**结论:**

NDMM患者浆细胞GPRC5D阳性率与1q21扩增及免疫状态有关。初诊时GPRC5D高表达可能预示患者对诱导治疗疗效不佳、预后不良。

## Abstract (en)

**Objective:**

To investigate GPRC5D expression on myeloma cells in newly diagnosed multiple myeloma（NDMM）patients and evaluate its prognostic significance.

**Methods:**

This study retrospectively analyzed the clinical data of 65 patients with NDMM treated at the Affiliated Hospital of Xuzhou Medical University from April 2023 to April 2024. The expression of GPRC5D on the surface of myeloma cells in all patients was detected with flow cytometry before induction therapy, and patients were stratified into high and low GPRC5D expression groups based on the median GPRC5D positivity rate. Clinical characteristics, immune status, treatment response after induction therapy, and prognosis were compared between the two groups.

**Results:**

The median positive rate of GPRC5D in the plasma cells of 65 patients with NDMM was 32.68％. Based on this threshold, patients were categorized into the high（33 cases, GPRC5D positive rate ≥ 32.68％）and low（32 cases, GPRC5D positive rate <32.68％）GPRC5D expression groups. Compared with the low GPRC5D expression group, the high GPRC5D expression group demonstrated a higher proportion of 1q21 gain（78.8％ *vs* 43.8％, *P*＝0.004）, a higher incidence of immunoparesis involving ≥2 uninvolved immunoglobulins（87.9％ *vs* 62.5％, *P*＝0.018）, and severe immunoparesis（59.4％ *vs* 33.3％, *P*＝0.046）. Further, CD16^+^CD56^+^ cell levels were lower in the high GPRC5D expression group［（16.60±8.70）％ *vs*（27.78±15.78）％, *P*＝0.005］. No significant difference was observed in the overall response rate between the high and low GPRC5D expression groups（78.8％ *vs* 93.8％, *P*＝0.165）. However, the high GPRC5D expression group exhibited a significantly lower rate of achieving very good partial remission or better（42.4％ *vs* 78.2％, *P*＝0.003）and a lower MRD negativity rate（30.0％ *vs* 68.8％, *P*＝0.002）. Compared with the low GPRC5D expression group, patients with high expression demonstrated a significantly shorter median progression-free survival（11.2 months *vs* not reached, *P*＝0.002）, whereas the median overall survival was not reached in either group, with no statistically significant difference（*P*＝0.069）.

**Conclusion:**

The GPRC5D positivity rate in the plasma cells of patients with NDMM is associated with 1q21 gain and immune status. High GPRC5D expression at diagnosis may predict poor response to induction therapy and an unfavorable prognosis.

多发性骨髓瘤（MM）是常见的血液系统恶性肿瘤，尽管蛋白酶体抑制剂（PI）、免疫调节剂（IMiD）、单克隆抗体和自体造血干细胞移植（auto-HSCT）等疗法显著改善了MM患者的预后，患者的中位生存期显著延长，但疾病仍不可治愈[1]–[3]。由于MM的临床表现、生物学特征和疗效存在差异，探索新诊断MM（NDMM）患者更实用、准确的预后指标很有必要。

G蛋白偶联受体C类第5组成员D（G-protein-coupled receptor family C group 5 member D，GPRC5D）是G蛋白偶联受体C家族的一员，是一种孤儿受体，具有七次跨膜结构[4]。GPRC5D在正常组织中表达有限，但在MM细胞中特异性高表达，被多项研究证实是有潜力的MM免疫治疗靶点，且可能适合作为定量检测肿瘤负荷的标志物[5]–[7]。Atamaniuk等[8]的研究显示，GPRC5D mRNA水平与MM患者的总生存（OS）期相关，Smith等[7]的研究提示，GPRC5D mRNA高水平患者表现出更短的无进展生存（PFS）期，但也有研究显示GPRC5D表达水平与患者的预后无显著相关性[7]–[9]。GPRC5D表达水平在MM中的预后价值仍存在争议。目前尚无研究探讨GPRC5D的表达水平与中国NDMM患者预后的相关性。本研究分析了65例NDMM患者的临床资料，旨在探讨GPRC5D在NDMM患者骨髓瘤细胞上的表达情况并评估其预后价值。

## 病例与方法

1. 病例：选取2023年4月至2024年4月在徐州医科大学附属医院确诊的65例MM患者为研究对象，MM的诊断标准与国际骨髓瘤工作组（IMWG）相符[10]。纳入患者在初诊时均进行流式细胞术检测、有明确的浆细胞GPRC5D检测结果、具有与诊断及疗效评估相关的检查指标并能获得完整的随访资料。冒烟型骨髓瘤、浆细胞白血病及合并其他恶性肿瘤的患者被排除。本研究经徐州医科大学附属医院伦理委员会批准（伦理批号：XYFY2024-KL613-01）。

2. 一般资料：通过电子病历系统回顾性收集NDMM患者的临床资料和实验室数据，包括年龄、性别、免疫球蛋白、HGB、白蛋白、β_2_微球蛋白、乳酸脱氢酶、血肌酐、血清钙、骨髓中单克隆浆细胞比例、是否合并髓外病变（EMD）、细胞遗传学异常、治疗方案、疾病进展时间和患者生存时间等。免疫麻痹定义为至少1种未受累免疫球蛋白低于正常值下限，重度免疫麻痹定义为至少1种未受累免疫球蛋白低于正常值下限50％以上[11]。

3. 流式细胞术检测GPRC5D表达：采集NDMM患者的骨髓标本，应用裂解液去除红细胞。PBS洗涤细胞后，应用CD45、CD38、CD138、BCMA、GPRC5D抗体混合液染色。随后用流式细胞术检测，先以FSC/SSC设门去除细胞碎片，再以CD45/CD38设门，圈定CD45^low^CD38^high^的恶性浆细胞群，分析骨髓瘤细胞上GPRC5D的表达情况。

4. FISH检测：FISH被用于检测细胞遗传学异常。采集NDMM患者的骨髓样本，使用CD138磁珠纯化浆细胞并进行FISH检测。使用的细胞遗传学探针包括gain（1q21）、del（13q14）、del（17p）和IgH易位（IgH/CCND1、IgH/FGFR3、IgH/MAF）。根据欧洲骨髓瘤网络（EMN）的标准，易位探针的阳性阈值被定义为10％，拷贝数探针的阳性阈值被定义为20％[12]。

5. 治疗方案与疗效：所有NDMM患者均接受至少含1种新药的诱导治疗方案，包括以PIs、IMiDs和达雷妥尤单克隆抗体为基础的方案，4个疗程后参照IMWG标准评估患者疗效[10]，包括严格意义的完全缓解（sCR）、完全缓解（CR）、非常好的部分缓解（VGPR）、部分缓解（PR）、疾病稳定（SD）和疾病进展（PD）。总体反应率（ORR）为CR率、VGPR率及PR率之和。同时采用二代流式细胞术（NGF）评估微小残留病（MRD），敏感度至少为10^−5^。

6. 随访：通过电话或查阅电子病历的方法对所有患者进行随访，随访截止日期为2024年12月31日。OS期定义为自患者确诊至因任何原因死亡的时间，PFS期定义为自患者确诊至疾病进展或因任何原因死亡的时间。末次随访时未发生复发、进展或死亡事件的患者按照截尾数据处理。

7. 统计学处理：使用R软件（version 4.4.1）进行统计学分析。符合正态分布的连续变量以*x*±*s*表示，非正态分布的连续变量以*M*（*Q*_1_，*Q*_3_）表示，使用独立样本*t*检验或Mann-Whitney *U*检验进行组间比较。分类变量以例数（百分比）表示，使用卡方检验或Fisher确切概率法进行组间比较。采用Kaplan-Meier法绘制生存曲线，Log-rank检验比较组间生存差异，双侧*P*<0.05为差异有统计学意义。

## 结果

1. 基本临床特征：本研究共纳入符合IMWG标准的NDMM患者65例，其中男40例（61.5％），女25例（38.5％）。中位年龄62（58～70）岁，65岁及以上26例（40.0％）。IgG型22例（33.8％），IgA型25例（38.5％），IgD型1例（1.5％），IgM型1例（1.5％），轻链型15例（23.2％），不分泌型1例（1.5％）。明确修订的国际分期系统（R-ISS）分期的患者54例，其中R-ISS Ⅲ期11例（20.4％）。10例（15.4％）患者骨髓浆细胞比例≥50％，15例（23.1％）患者合并EMD。FISH结果异常61例（93.8％），其中17p缺失9例（13.8％），1q21扩增40例（61.5％），13q14缺失35例（53.8％），IgH重排56例（86.2％）。30例患者进一步检测t（4;14）、t（11;14）、t（14;16），分别检出6例（20.0％）、6例（20.0％）和1例（3.3％）（表1）。

**表1:** 新诊断多发性骨髓瘤患者浆细胞GPRC5D低表达组和高表达组患者临床特征比较

临床特征	GPRC5D低表达组（32例）	GPRC5D高表达组（33例）	*z/χ*^2^值	*P*值
浆细胞GPRC5D阳性率［％，*M*（*Q*_1_，*Q*_3_）］	8.54（3.41，15.57）	78.63（59.94，95.65）	−8.90	<0.001
年龄［例（％）］			2.01	0.156
<65岁	22（68.8）	17（51.5）		
≥65岁	10（31.2）	16（48.5）		
性别［例（％）］			0.44	0.505
男	21（65.6）	19（57.6）		
女	11（34.4）	14（42.4）		
免疫分型［例（％）］			–	0.495
IgG型	11（34.4）	11（33.3）		
IgA型	10（31.3）	15（45.5）		
IgD型	1（3.1）	0（0）		
IgM型	0（0）	1（3.0）		
轻链型	9（28.1）	6（18.2）		
不分泌型	1（3.1）	0（0）		
R-ISS分期［例（％）］^a^			–	0.762
Ⅰ期	2（8.3）	4（13.3）		
Ⅱ期	16（66.7）	21（70.0）		
Ⅲ期	6（25.0）	5（16.7）		
HGB［例（％）］			0.13	0.722
<100 g/L	18（56.2）	20（60.6）		
≥100 g/L	14（43.8）	13（39.4）		
白蛋白［例（％）］			0.14	0.710
<30 g/L	7（21.9）	6（18.2）		
≥30 g/L	25（78.1）	27（81.8）		
β_2_微球蛋白［例（％）］			1.93	0.165
<5.5 mg/L	18（56.3）	24（72.7）		
≥5.5 mg/L	14（43.7）	9（27.3）		
血肌酐［例（％）］			0.15	0.699
<177 µmol/L	26（81.2）	28（84.9）		
≥177 µmol/L	6（18.8）	5（15.1）		
血清钙［例（％）］			0.00	0.966
<2.65 mmol/L	28（87.5）	30（90.9）		
≥2.65 mmol/L	4（12.5）	3（9.1）		
乳酸脱氢酶［例（％）］			0.05	0.821
<250 U/L	25（78.1）	25（75.8）		
≥250 U/L	7（21.9）	8（24.2）		
骨髓瘤细胞比例［例（％）］		0.08	0.771
<50％	28（87.5）	27（81.8）		
≥50％	4（12.5）	6（18.2）		
合并髓外病变［例（％）］	8（25.0）	7（21.2）	0.13	0.717
细胞遗传学异常［例（％）］				
17p缺失	4（12.5）	5（15.2）	0.00	1.000
1q21扩增	14（43.8）	26（78.8）	8.43	0.004
13q14缺失	15（46.9）	20（60.6）	1.23	0.267
IgH重排	27（84.4）	29（87.9）	0.00	0.960
t（4;14）^b^	4（30.8）	2（11.8）	–	0.360
t（11;14）^b^	3（23.1）	3（17.7）	–	1.000
t（14;16）^b^	1（7.7）	0（0）	–	0.433

**注** R-ISS：修订的国际分期系统；^a^共54例患者可评估；^b^共30例患者可评估；–：应用Fisher确切概率法

浆细胞GPRC5D阳性率的中位值为32.68％（9.79％～78.63％）。依据中位数将65例NDMM患者分为GPRC5D高表达组（33例）和低表达组（32例），比较两组患者的临床特征。结果显示，与GPRC5D低表达组相比，GPRC5D高表达组的1q21扩增比例较高（78.8％对43.8％，*P*＝0.004）。两组患者年龄、性别、R-ISS分期、白蛋白、β_2_微球蛋白、乳酸脱氢酶、骨髓瘤细胞比例、EMD等特征的差异均无统计学意义（*P*值均>0.05）（表1）。

2. GPRC5D表达情况与淋巴细胞亚群及未受累免疫球蛋白水平的相关性：对NDMM患者外周血进行淋巴细胞亚群检测，比较GPRC5D高表达组与低表达组患者淋巴细胞水平的差异。结果显示，GPRC5D高表达组患者的CD16^+^CD56^+^细胞水平低于GPRC5D低表达组［（16.60±8.70）％对（27.78±15.78）％，*P*＝0.005］，其余指标的差异均无统计学意义（*P*值均>0.05）（表2）。

**表2:** 新诊断多发性骨髓瘤患者浆细胞GPRC5D低表达组和高表达组患者淋巴细胞亚群及未受累免疫球蛋白水平比较

变量	GPRC5D低表达组（32例）	GPRC5D高表达组（33例）	统计量	*P*值
淋巴细胞亚群^a^				
CD19^+^［％，*M*（*Q*_1_，*Q*_3_）］	4.65（1.79，6.50）	4.32（1.41，9.30）	*z*＝−0.45	0.651
CD3^+^［％，*M*（*Q*_1_，*Q*_3_）］	70.89（60.05，75.36）	74.10（66.80，84.52）	*z*＝−1.40	0.161
CD3^+^CD8^+^［％，*M*（*Q*_1_，*Q*_3_）］	24.58（20.29，32.46）	28.14（23.03，42.22）	*z*＝−1.25	0.210
CD3^+^CD4^+^（％，*x*±*s*）	33.72±13.75	34.75±13.67	*t*＝−0.25	0.804
CD16^+^CD56^+^（％，*x*±*s*）	27.78±15.78	16.60±8.70	*t*＝2.95	0.005
CD4/CD8（*x*±*s*）	1.55±0.90	1.31±0.74	*t*＝0.96	0.345
未受累免疫球蛋白［g/L，*M*（*Q*_1_，*Q*_3_）］				
IgG	5.48（4.14，6.76）	3.71（3.47，4.62）	*z*＝−3.13	0.002
IgA	0.43（0.20，0.81）	0.17（0.11，0.35）	*z*＝−2.19	0.028
IgM	0.25（0.15，0.34）	0.16（0.11，0.25）	*z*＝−1.66	0.097
免疫麻痹［例（％）］				
≥1种未受累免疫球蛋白	27（84.4）	32（97.0）	*χ*²＝1.76	0.185
≥2种未受累免疫球蛋白	20（62.5）	29（87.9）	*χ*²＝5.64	0.018
重度免疫麻痹	9（33.3）	19（59.4）	*χ*²＝3.98	0.046

**注** ^a^淋巴细胞亚群检测例数为44例

与GPRC5D低表达组患者相比，GPRC5D高表达组患者的多克隆IgG水平（3.71 g/L对5.48 g/L，*P*＝0.002）和多克隆IgA水平（0.17 g/L对0.43 g/L，*P*＝0.028）较低，差异有统计学意义。多克隆IgM水平也存在类似结果，但差异无统计学意义（0.16 g/L对0.25 g/L，*P*＝0.097）。此外，GPRC5D高表达组患者出现≥2种未受累免疫球蛋白免疫麻痹比例（87.9％对62.5％，*P*＝0.018）和重度免疫麻痹比例（59.4％对33.3％，*P*＝0.046）高于GPRC5D低表达组患者（表2）。

3. GPRC5D与诱导治疗疗效的相关性：所有NDMM患者均接受了诱导治疗，接受1种新药方案治疗的患者13例（20％），接受≥2种新药方案的患者52例（80％），病程中26例（40％）患者接受auto-HSCT。GPRC5D高表达组和低表达组接受的诱导治疗方案及接受auto-HSCT患者占比差异均无统计学意义（*P*值均>0.05）。诱导治疗4个疗程后评估，NDMM患者的ORR为86.2％，其中GPRC5D高表达组的ORR为78.8％，而GPRC5D低表达组的ORR为93.8％，差异无统计学意义（*P*＝0.165）。GPRC5D高表达组疗效达到VGPR及以上者比例显著低于低表达组（42.4％对78.2％，*P*＝0.003）（表3）。此外，GPRC5D高表达组患者诱导治疗后MRD阴性率低于GPRC5D低表达组（30.0％对68.8％，*P*＝0.002）。

根据诱导治疗方案将NDMM患者分为两个亚组，分别比较接受1种和≥2种新药方案治疗的患者中，GPRC5D表达与疗效的相关性。结果显示，在接受同一组治疗方案的患者中，GPRC5D高表达组疗效达到VGPR及以上者比例较GPRC5D低表达组低（*P*值分别为0.021和0.048）（表3）。在GPRC5D低表达组中，接受1种新药方案和≥2种新药方案治疗患者的ORR分别为100.0％和92.3％（*P*＝1.000），疗效达到VGPR及以上患者占比分别为100.0％和73.1％（*P*＝0.296）。在GPRC5D高表达组患者中，接受1种、≥2种新药方案治疗患者的ORR分别为71.4％、80.8％（*P*＝0.623），疗效达到VGPR及以上患者占比分别为28.6％、46.2％（*P*＝0.670）。

**表3:** 新诊断多发性骨髓瘤患者浆细胞GPRC5D低表达组和高表达组患者疗效比较

疗效评价	GPRC5D低表达组（32例）	GPRC5D高表达组（33例）	*χ*^2^值	*P*值
全部患者［例（％）］				
CR	18（56.3）	10（30.3）		
VGPR	7（21.9）	4（12.1）		
PR	5（15.6）	12（36.4）		
SD	0（0.0）	1（3.0）		
PD	2（6.2）	6（18.2）		
ORR	30（93.8）	26（78.8）	1.92	0.165
CR+VGPR	25（78.2）	14（42.4）	8.63	0.003
1种新药方案［例（％）］^a^				
CR	4（66.7）	1（14.3）		
VGPR	2（33.3）	1（14.3）		
PR	0（0.0）	3（42.8）		
SD	0（0.0）	0（0.0）		
PD	0（0.0）	2（28.6）		
ORR	6（100.0）	5（71.4）	–	0.462
CR+VGPR	6（100.0）	2（28.6）	–	0.021
≥2种新药方案［例（％）］^b^				
CR	14（53.9）	9（34.6）		
VGPR	5（19.2）	3（11.6）		
PR	5（19.2）	9（34.6）		
SD	0（0.0）	1（3.8）		
PD	2（7.7）	4（15.4）		
ORR	24（92.3）	21（80.8）	0.66	0.416
CR+VGPR	19（73.1）	12（46.2）	3.91	0.048

注 CR：完全缓解；VGPR：非常好的部分缓解；PR：部分缓解；SD：疾病稳定；PD：疾病进展；ORR：总体反应率；^a^接受1种新药方案的患者共13例；^b^接受≥2种新药方案的患者共52例；–：应用Fisher确切概率法

4. 生存分析：65例NDMM患者的中位随访时间为12.5（95％ *CI*：11.7～14.4）个月，中位PFS期为16.5个月（95％ *CI*：11.8个月～未达到），中位OS期未达到。GPRC5D高表达、低表达组的1年PFS率分别为34.9％（95％ *CI*：21.2％～57.5％）、83.6％（95％ *CI*：71.4％～97.9％），差异有统计学意义（*P*<0.001）。GPRC5D高表达和低表达组患者的1年OS率差异无统计学意义［（81.0％（95％ *CI*：68.3％～96.1％对95.2％（95％ *CI*：86.6％～100％），*P*＝0.118］。GPRC5D高表达组患者较低表达组有更短的中位PFS期（11.2个月对未达到，*P*＝0.002）（图1A），而两组的中位OS期均未达到，差异无统计学意义（*P*＝0.069）（图1B）。

**图1:**
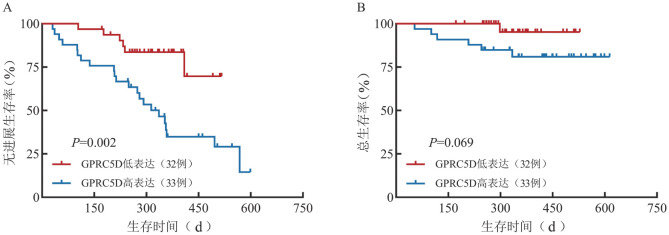
新诊断多发性骨髓瘤患者浆细胞GPRC5D低表达组和高表达组的无进展生存（A）和总生存（B）曲线

## 讨论

MM仍是一种不可治愈的恶性浆细胞疾病，具有复杂的遗传异质性[1]。传统的风险分层系统，如国际分期系统（ISS）和R-ISS，长期以来被广泛应用于MM患者的预后评估。这些分期系统基于临床和实验室参数，如白蛋白、β_2_微球蛋白、乳酸脱氢酶及细胞遗传学异常，为患者提供了初步的风险分层，较好地指导了临床。然而，新药时代的多项研究结果显示，这些分期系统对MM患者预后的预测价值不足，可能无法全面反映疾病的生物学复杂性和个体差异[13]–[14]。因此，寻找新的MM预后生物标志物对提高预后评估的准确性、指导个体化治疗策略、改善患者预后具有重要意义。

GPRC5D定位于染色体12p13.3，是一类孤儿G蛋白偶联受体，其内源配体未定，介导的信号传导机制及具体功能尚不明确[4]。在正常组织中，GPRC5D主要表达在皮肤角化组织和浆细胞上，而其他免疫细胞和健康组织几乎不表达。此外，相较于其他肿瘤细胞，GPRC5D特异性地高表达于MM细胞上[7],[15]。GPRC5D的特异性表达使其成为一个具有潜力的MM免疫治疗靶点，近年来，靶向GPRC5D的双特异性抗体、嵌合抗原受体T细胞等疗法在MM的治疗中展现了令人鼓舞的前景[5],[16]–[19]。

既往研究显示，骨髓MM细胞GPRC5D表达水平升高与较高的β_2_微球蛋白、原始浆细胞增多、13q14缺失、t（4;14）、t（11;14）、1q21扩增及高ISS分期相关[8]–[9]。本研究依据浆细胞GPRC5D阳性率的中位数将NDMM患者分为两组，比较两组患者临床特征的差异，结果显示，GPRC5D高表达组1q21扩增比例显著高于GPRC5D低表达组。1q21扩增是MM患者高危细胞遗传学异常之一，被多项MM预后分层系统列为MM的高危因素。伴有1q21扩增的患者常表现为更高的肿瘤负荷，更明显的终末器官损伤，且更易合并其他高危细胞遗传学异常。同时，1q21扩增与疾病进展和不良预后密切相关[20]–[22]。

淋巴细胞亚群是免疫系统的重要组成部分，对调节肿瘤免疫应答具有关键作用，其功能障碍可减弱机体对肿瘤细胞的清除作用，与MM的发生及发展密切相关[23]。本研究显示，GPRC5D高表达组患者的CD16^+^CD56^+^细胞即NK细胞水平明显低于GPRC5D低表达组。NK细胞是先天免疫系统的细胞毒性淋巴细胞，作用较为广泛，既能直接杀伤肿瘤细胞，又能产生促炎细胞因子，间接增强T细胞介导的免疫反应[24]。有研究显示，MM患者NK细胞数量减少、活性减低，使免疫监视功能受损，导致疾病进展[25]。多克隆免疫球蛋白降低即免疫麻痹普遍存在于NDMM患者中，被认为是免疫系统活性降低的标志，既往研究报道，免疫麻痹的发生、严重程度及持续时间与MM患者的不良预后相关[11],[26]。本研究同样发现，GPRC5D高表达组患者多克隆免疫球蛋白受抑制的数量更多、程度更深，诱导治疗后达到PR以上的患者比例更低，具有更短的PFS期。

本研究进一步探索了GPRC5D表达与诱导治疗后疗效的相关性。GPRC5D高表达组患者诱导治疗后达到VGPR及以上的比例显著低于GPRC5D低表达组患者，ORR也更低，尽管差异无统计学意义。在接受1种新药方案治疗的亚组和2～3种新药方案治疗的亚组中，GPRC5D高表达组患者疗效达到VGPR及以上者比例均更低。值得注意的是，初始GPRC5D低表达组患者接受1种新药方案治疗的ORR及VGPR/CR率均达到100％，提示因高龄、肾功能受损严重或基础状态差等因素无法耐受更强烈的诱导方案的患者中，更推荐应用只含有1种新药的方案诱导治疗。这一发现对于指导临床实践、优化治疗方案及减少不必要的治疗强度具有重要意义，特别是在需要平衡治疗有效性与患者耐受性的复杂情况下。

GPRC5D与MM预后的相关性仍存在争议。Atamaniuk等[8]发现骨髓GPRC5D mRNA表达水平与MM患者的OS显著相关。该研究采用实时聚合酶链反应（RT-PCR）技术对48例MM患者的骨髓进行检测，根据GPRC5D mRNA表达量的中位数分为两组，发现高于中位数的患者OS期明显更短。Smith等[7]对CoMM pass队列中765例MM患者进行分析，结果显示，GPRC5D mRNA表达量高于中位数的患者具有较短的PFS期。但最新一项研究显示，GPRC5D表达水平与MM患者的PFS和OS没有显著相关性[9]。而在本研究中，我们的结果表明，GPRC5D高表达组较GPRC5D低表达组患者有更低的1年PFS率及OS率，同样观察到较短的PFS期及OS期。OS期的差异无统计学意义可能与随访时间较短及样本量偏小有关。GPRC5D表达水平对NDMM患者长期预后的影响还有待进一步观察。

总之，本研究观察到NDMM患者骨髓瘤细胞GPRC5D表达水平与1q21扩增、免疫状态密切相关，初诊时GPRC5D高表达可能预示患者对诱导治疗反应不佳及预后不良。然而，由于本研究样本量不大，随访时间较短，并未对浆细胞GPRC5D阳性率预测预后的最佳阈值进行分析。后续需要增加样本量、延长随访时间以进一步验证GPRC5D表达情况对NDMM患者预后的影响。
